# Magnetic resonance-guided motorized transcranial ultrasound system for blood-brain barrier permeabilization along arbitrary trajectories in rodents

**DOI:** 10.1186/s40349-015-0044-5

**Published:** 2015-12-24

**Authors:** Rémi Magnin, Fabien Rabusseau, Frédéric Salabartan, Sébastien Mériaux, Jean-François Aubry, Denis Le Bihan, Erik Dumont, Benoit Larrat

**Affiliations:** UNIRS, Neurospin, I2BM, Direction des Sciences du Vivant, Commissariat à l’Energie Atomique et aux Energies Alternatives, Bâtiment 145, 91191 Gif sur Yvette, France; Image Guided Therapy, 4 allée du doyen Brus, 33600 Pessac, France; CNRS UMR 7587, INSERM U979, ESPCI ParisTech, Institut Langevin Ondes et Images, 1 rue Jussieu, 75005 Paris, France

**Keywords:** Blood-brain barrier permeabilization, Focused ultrasound, High-field magnetic resonance imaging, Drug delivery to rodent brains

## Abstract

**Background:**

Focused ultrasound combined with microbubble injection is capable of locally and transiently enhancing the permeability of the blood-brain barrier (BBB). Magnetic resonance imaging (MRI) guidance enables to plan, monitor, and characterize the BBB disruption. Being able to precisely and remotely control the permeabilization location is of great interest to perform reproducible drug delivery protocols.

**Methods:**

In this study, we developed an MR-guided motorized focused ultrasound (FUS) system allowing the transducer displacement within preclinical MRI scanners, coupled with real-time transfer and reconstruction of MRI images, to help ultrasound guidance. Capabilities of this new device to deliver large molecules to the brain on either single locations or along arbitrary trajectories were characterized in vivo on healthy rats and mice using 1.5 MHz ultrasound sonications combined with microbubble injection. The efficacy of BBB permeabilization was assessed by injecting a gadolinium-based MR contrast agent that does not cross the intact BBB.

**Results:**

The compact motorized FUS system developed in this work fits into the 9-cm inner diameter of the gradient insert installed on our 7-T preclinical MRI scanners. MR images acquired after contrast agent injection confirmed that this device can be used to enhance BBB permeability along remotely controlled spatial trajectories of the FUS beam in both rats and mice. The two-axis motor stage enables reaching any region of interest in the rodent brain. The positioning error when targeting the same anatomical location on different animals was estimated to be smaller than 0.5 mm. Finally, this device was demonstrated to be useful for testing BBB opening at various acoustic pressures (0.2, 0.4, 0.7, and 0.9 MPa) in the same animal and during one single ultrasound session.

**Conclusions:**

Our system offers the unique possibility to move the transducer within a high magnetic field preclinical MRI scanner, thus enabling the delivery of large molecules to virtually any rodent brain area in a non-invasive manner. It results in time-saving and reproducibility and could be used to either deliver drugs over large parts of the brain or test different acoustic conditions on the same animal during the same session, therefore reducing physiological variability.

## Background

Focused ultrasound (FUS) is an emerging field [1] which has shown promising clinical results in a wide range of indications, such a prostate cancer [2, 3], liver carcinomas [4, 5], uterine fibroid [6, 7], or bone metastasis [8, 9]. In the brain, FUS has shown its potential in thermal thalamotomy for neuropathic pain [10] or essential tremor [11].

In the last decade, preclinical studies demonstrated that burst sonications combined with intravenous injection of microbubbles were able to disrupt the blood-brain barrier (BBB) locally, transiently, and without damages, allowing the delivery to brain tissues of large molecules which cannot normally access the central nervous system (CNS) because of their size [12, 13]. Many feasibility studies have investigated the capability of FUS-induced BBB disruption to massively enhance the delivery of a wide variety of therapeutic agents such as anticancer drugs [14–16], anti-amyloid antibodies [17–19], siRNA, or nanoparticles [20]. Recently, repeated application of BBB permeabilization protocols has also demonstrated its ability to reduce the amyloid plaque load in mice model of Alzheimer’s disease, without any use of therapeutic drug [21, 22]. A growing number of studies have been exploring the optimal parameters for BBB disruption such as the influence of ultrasound parameters [23–26] and microbubble properties [27, 28], or the physiologic state of the animals (temperature, anesthesia) [29] and the maximum gap size obtained in the vascular walls and the closure dynamics [30]. Nevertheless, the exact mechanism leading to BBB permeabilization is still not fully understood. To be later transferred to humans, or even disseminated as a tool for pharmacological proofs of concept, there is a need for further investigating BBB disruption in preclinical settings. The variety of brain disease models available on rodents makes them good candidates for these studies.

To carry out these studies, magnetic resonance imaging (MRI) monitoring of FUS experiment is widely used. Indeed, the physical effects of ultrasound on the brain can be directly visualized using dedicated sequences such as acoustic radiation force imaging (ARFI) for ultrasound localization and in situ acoustic pressure measurements [31, 32], or thermometry sequences to measure temperature changes during thermal applications. In the meantime, standard sequences can be used for anatomical targeting and radiological assessment of desirable and undesirable tissue damages due to ultrasound application. Planning and monitoring the experiment is of importance in order to avoid known risks such as edema or hemorrhages due to excessive acoustic pressure responsible for inertial cavitation of circulating microbubbles. In the case of BBB permeabilization experiments, the injection of MRI contrast agents, such as gadolinium (Gd) chelates, also provides evidence of the disruption, as well as accurate characterization of the permeability [33]. In addition, it can be valuable to have a real-time reconstruction and display of the MR images to plan and monitor the experiment with a feedback on the FUS system.

To carry out MR-guided BBB permeabilization experiments, a number of features are necessary. It requires a system to hold the head to avoid movement during MR acquisitions. A radiofrequency (RF) coil is also needed for MR imaging. Finally, the possibility to position precisely the focal spot in the brain is essential. In this purpose, a good solution is to use MR-compatible motors allowing a displacement of the transducer [34] within the magnet. It enables a fast and accurate repositioning of the transducer, and moving the focal spot during sonications also allows treating larger regions [22]. Unfortunately, due to the limited space offered by preclinical MRI scanners and the MR compatibility problems arising at high magnetic fields, these systems only operate within 3-T clinical MRI scanners. Thus, one cannot benefit from the advantages offered by dedicated preclinical high magnetic field MRI scanners. Moreover, it can be of great interest to have a system which can adapt to different species to widen the experimental possibilities.

In this study, such a MR-compatible motorized system operating in a 7-T preclinical MRI scanner was developed and validated. Efforts were put to develop interchangeable beds integrating the RF coil, ear bars, and a bite bar, so that the setup could be used for different species (rats and mice). In addition, real-time transfer and reconstruction of MR images was implemented to ensure a good monitoring of the experiment.

Finally, in vivo BBB opening along arbitrary trajectories in the rodent brain under preclinical high magnetic field (7 T) MR guidance is demonstrated. The capability of the system to perform BBB opening experiments under several different acoustic conditions in the same animal at once is also demonstrated.

## Methods

### Ultrasound transducer

**Fig. 1:**
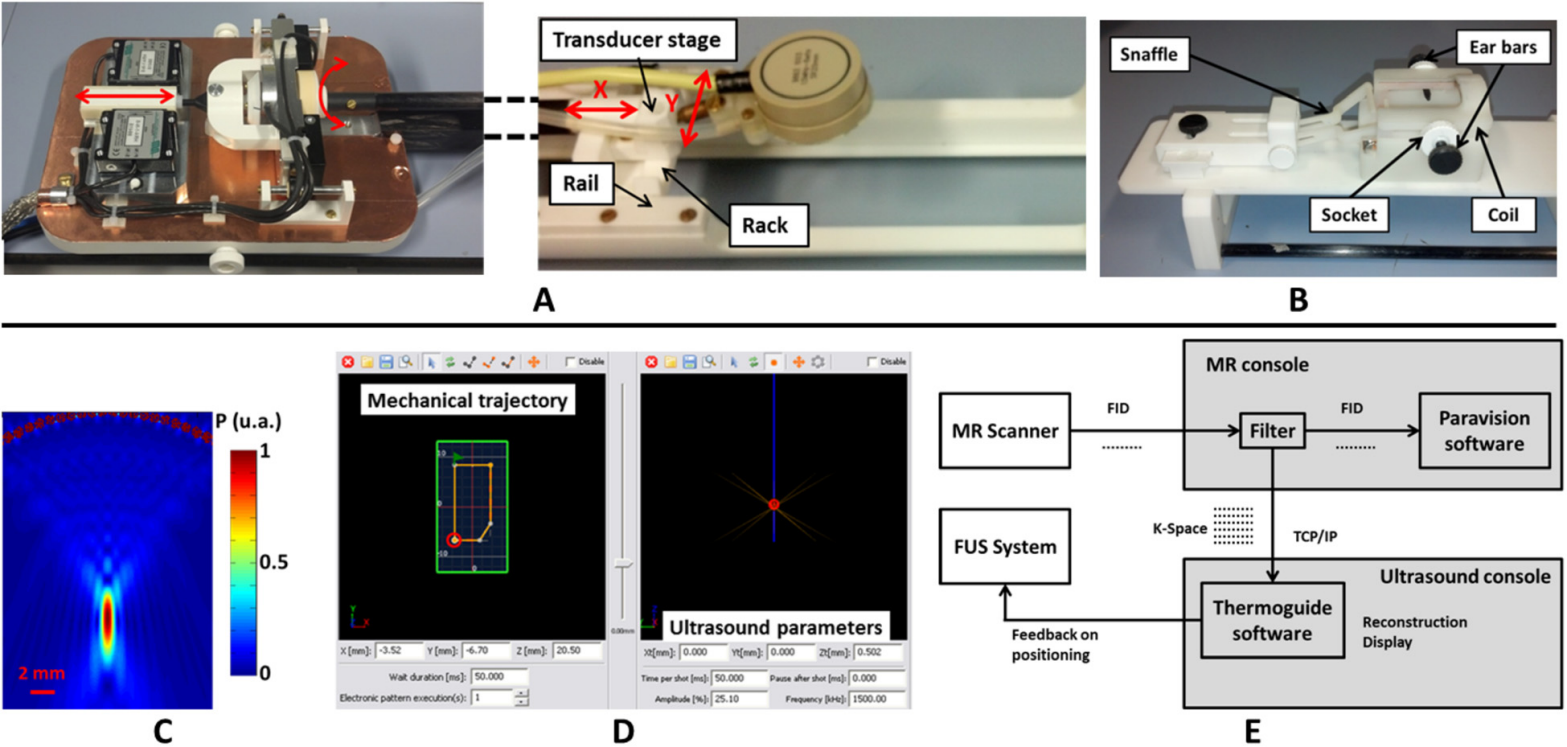
Overview of the setup. **a** On the left, the block with the motors stays outside the magnet, and the movement is transmitted to the transducer on the right via a glass fiber stick. The cradle is holed to welcome an interchangeable bed which is plugged into it. **b** An interchangeable bed including a plastic head holder with ear bars and bite bar and a dedicated ultrasound transparent coil. **c** Simulation of the acoustic pressure field generated by the 1.5-MHz monoelement transducer A. **d** A screen print of the software used to drive the motors and the ultrasound. **e** A scheme of the real-time data transmission and reconstruction

For the first experiments aimed at testing the motorization in vivo, a single-element MR-compatible focused transducer (transducer A, diameter 25 mm, focal depth 20 mm, Imasonic, France) resonating at 1.5 MHz was used and is pictured in Fig. 3 [31]. In order to be able to target deeper regions of the brain, a phased array annular transducer with eight channels and a central frequency of 1.5 MHz was also designed and purchased from Imasonic, France (Fig. 1a). It consisted of a spherically curved phased array of concentric rings made of piezoelectric composite embedded in a plastic mount (transducer B, diameter 30 mm, geometrical focal depth 20 mm). The use of several elements allowed electronic steering of the ultrasound beam in depth. Both transducers were coupled to the rodent head with a water balloon filled with deionized and degassed water and closed by a thin latex membrane. The ultrasounds were shot from top to down.

The balloon was connected to a water degassing system. Degassed, deionized water circulated prior to the experiments in order to eliminate air bubbles trapped in the balloon. The circulation was stopped during MR acquisitions to avoid artifacts induced by the water movement. The hydrostatic pressure in the balloon could be adjusted manually to inflate or deflate the balloon and ensure proper coupling with the animal head.

### In vitro acoustic calibration

The output pressure of the transducers and the full width at half maximum (FWHM) of the focal spot were measured in a degassed water tank, using a 200-μm calibrated hydrophone (HGL-0200, preamplifier AH-2020, Onda Corporation, USA) mounted on a micrometric 3D positioning stage. The transducers were excited with a 15-cycle pulse and 1-Hz repetition frequency. The peak negative pressure at the focus was recorded as a function of electrical power in order to provide a calibration curve for each transducer. In addition, the acoustic fields generated by the transducers were simulated using Fields II software [35, 36] (see Fig. 1c). In the water tank, the FWHM were measured to be respectively 1.2*1.2*5.8 ± 0.1 mm for transducer A and 1.2*1.2*6.6 ± 0.1 mm for transducer B.

### MR-compatible motorization

To be able to precisely position the ultrasound focal spot in the rodent brain in 3D, the transducer holder could be moved mechanically in *xy* plane by motors, in addition to axial electronic steering along *z* axis for transducer B (see Fig. 1a).

Piezoelectric step motors have been installed outside the magnet and embedded in a Faraday cage to avoid emission of RFs. A glass fiber stick enabled the transmission of the motor displacement to the transducer (see Fig. 1a). This experimental setup combined MR compatibility at high magnetic field with a high degree of compactness.

For head/foot translation, the stick was linked via a ball joint to a block coated with ceramic stripe on its edges. Two identical motors (HR4, Nanomotion, Yoqneam, Israel) were set on each side of the block (Fig. 1a, left). Ceramic finger tips on the motors moved the block by pressing the ceramic stripe, inducing a translation of the transducer stage. The minimum step size was 0.005 millimeters (mm), and the total range was 27 mm. The measurement of the transducer position was achieved by an embedded optical system, consisting of one optical stripe attached under the block (MV 65-40 EP, Numerik Jena, Germany), facing a diode optical encoder (LIK 21, Numerik Jean, Germany). For absolute measurement, two references were located on the stripe and were detected during a motor initialization step at the beginning of each experiment. Further displacements were calculated with respect to these references.

Left/right translation was achieved with a plastic disk coated with ceramic and surrounding the glass fiber stick (Fig. 1a, left). A rotation of the disk was induced by two identical motors (HR2, Nanomotion, Yoqneam, Israel) set on each side of the disk and vibrating in same phases. The rotation was then converted to a translation with a rack and pinion system positioned on the transducer stage (Fig. 1a, right). The minimum step size was 0.0087 mm, for a total displacement range of 15 mm. An optical sensor (RIK 4, Numerik Jena, Germany) measured the displacement and took advantage of one reference stripe (RS 29/16/900, Numerik Jena, Germany) in order to estimate the absolute position.

The transducer stage consisted of a rectangular plastic plate guided by rails along the cradle. On its top, a mobile rack was guided in a perpendicular direction to convert the rotation of the glass fiber stick into a translation. The transducer holder was linked to its stage with a ball joint, allowing freedom of movement in the three directions for more efficient coupling to the rodent head.

### Interchangeable beds

To facilitate the installation of the animals outside the magnetic field, interchangeable beds were designed that can be unplugged from the rest of the MRI cradle. Two switchable beds were designed, one for mice and one for rats and made of polyethylene terephthalate (PETP).

Each bed included a plastic holder made of ABS resin to maintain the animal head during MRI acquisitions, an access for gas anesthetics (inflow and outflow tubes), and an MR RF coil (see Fig. 1b) and its corresponding tuning and matching circuit. Ear bars were attached on each side of the head holder with specially designed sockets. The sockets could adapt to the head size of the animals. In front of the head holder, a plastic mount held a bite bar or snaffle and another stick which could be positioned above the animal nose. Both could be adjusted in length and height, and the holder could also be moved perpendicular to the cradle to ensure a good adaptability.

### Integrated radiofrequency MRI coils

For an extended access of the transducer to the animal head while maximizing the SNR on the brain, several coil geometries were investigated. The simplest and more robust one is a single loop coil, whose diameter was wide enough for the ultrasound beam to pass through it and for extensive displacement of the transducer above the head.

The size of the RF resonator was adapted for each species: the loop diameter was tighter for the mice in order to maximize the filling factor. The resonators were made of copper stripes mounted on the head holder itself, at a resonance frequency of 300 MHz corresponding to the Larmor frequency of protons at 7 T. They were connected to a balanced circuit fixed under the bed, which allowed fine-tuning and matching of the resonance frequency via variable capacitors. RF transmission from and to Bruker amplifiers was made via a semi-rigid coaxial cable suitable for high power (1000 W), which laid under the bed. Capacitor adjustment from outside the magnet was carried out by specially designed plastic screwdrivers.

### Dedicated system control software

The dedicated Thermoguide® software (Image Guided Therapy, Pessac, France; see Fig. 1d) was installed on the ultrasound console. It allowed driving remotely the transducer and the motors by setting up trajectories and then sending them for execution to the FUS electronics. A trajectory is composed of segments defining straight displacements. Ultrasound can be shot along these segments.

On the mechanical trajectory panel (Fig. 1d), one could manually draw any arbitrary trajectory or define it more accurately by entering the coordinates of the segments. The motor speed can be tuned along each segment. The electronic trajectory panel (Fig. 1d) allowed controlling every ultrasound parameter (frequency, shot duration, pulse repetition frequency, amplitude) and the steering in depth if possible.

Once the trajectory was programmed, it was sent to the generator via an Ethernet connection and stored in a buffer before execution. The number of repetitions of this trajectory and the pause between two repetitions could be chosen, as well as different triggering options: no trigger, a trigger before each trajectory, or a trigger on every shot.

### Real-time data transfer and monitoring

**Fig. 2:**
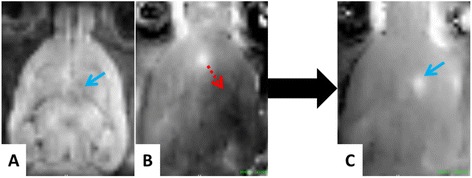
ARFI imaging and focal spot positioning procedure. Step 1: an anatomy image and an ARFI image are acquired and reconstructed in real time. Step 2: the target is chosen on the anatomy image (**a**, *blue arrow*), while the current position of the focal spot is determined using the ARFI phase image (**b**). Step 3: a feedback on the motors allows reaching the previously defined target location (**b**, *red arrow*). Step 4: the phase of an ARFI image acquired after the transducer displacement allows verifying that the desired target location has been reached (**c**, *blue arrow*)

A pipeline filter implemented on the MRI console running Paravision® software (Bruker, Germany) allowed real-time reconstruction and visualization of acquired images directly on the ultrasound console (Fig. 1e). During image acquisition, the filter intercepted the raw K-space data and sent them along with a header summarizing all acquisition parameters to the ultrasound console through a TCP/IP protocol. This real-time pipeline was successfully tested for all standard Bruker MRI sequences. Specific data processing could be applied in real time to the MR images, such as a temperature estimation or thermal dose measurement for thermometry experiments. In addition, a specific plug-in for the Thermoguide® software was developed to select the target location for BBB permeabilization on MR anatomical images. Then, the ultrasound focal spot position was retrieved either automatically or manually on phase images acquired with MR acoustic radiation force imaging (MR-ARFI) sequence, and a motor feedback finally moved the transducer to its target position (see Fig. 2).

### MRI acquisitions

All MRI acquisitions were performed in a 7-T preclinical scanner (Pharmascan, Bruker, Ettlingen, Germany) equipped with a 9-cm inner diameter 740-mT/m gradient insert.

In order to locate the ultrasound beam in vivo, an ARFI sequence was set by adding two sinusoidal motion-sensitizing gradients to a multi-slice multi-echo (MSME) sequence, synchronized with ultrasound bursts so that the acquired phase image is proportional to acoustic intensity [31]. The following parameters were used: echo time (TE)/repetition time (TR) = 28/1080 milliseconds (ms), spatial resolution = 0.5 × 0.5 × 2 mm^3^, matrix size = 64 × 64 × 5, number of averages = 2, duration of motion-encoding gradients = 8 ms, duration of ultrasound bursts = 4 ms, and total acquisition time = 2.5 min.

After BBB disruption protocol, a T_1_-weighted (T_1_w) MSME sequence (TE/TR = 8.3/300 ms, spatial resolution = 0.125 × 0.125 × 1 mm^3^, matrix size = 256 × 256 × 10, 10 averages, acquisition time = 6.5 min) was acquired to detect Gd chelates delivered to brain tissues due to enhanced BBB permeability.

To control the safety of the BBB disruption protocol, especially to detect the potential presence of hemorrhages or edema at the disruption site, a T_2_-weighted (T_2_w) image was acquired at the end of the experiment, using a Rapid Acquisition with Refocused Echoes (RARE) sequence, with the following parameters: TE_effective_/TR = 30/3800 ms, spatial resolution = 0.250 × 0.250 × 0.5 mm^3^, matrix = 128 × 120 × 32, RARE factor = 8, 8 averages, acquisition time = 7.5 min.

### BBB permeabilization protocols

**Fig. 3:**
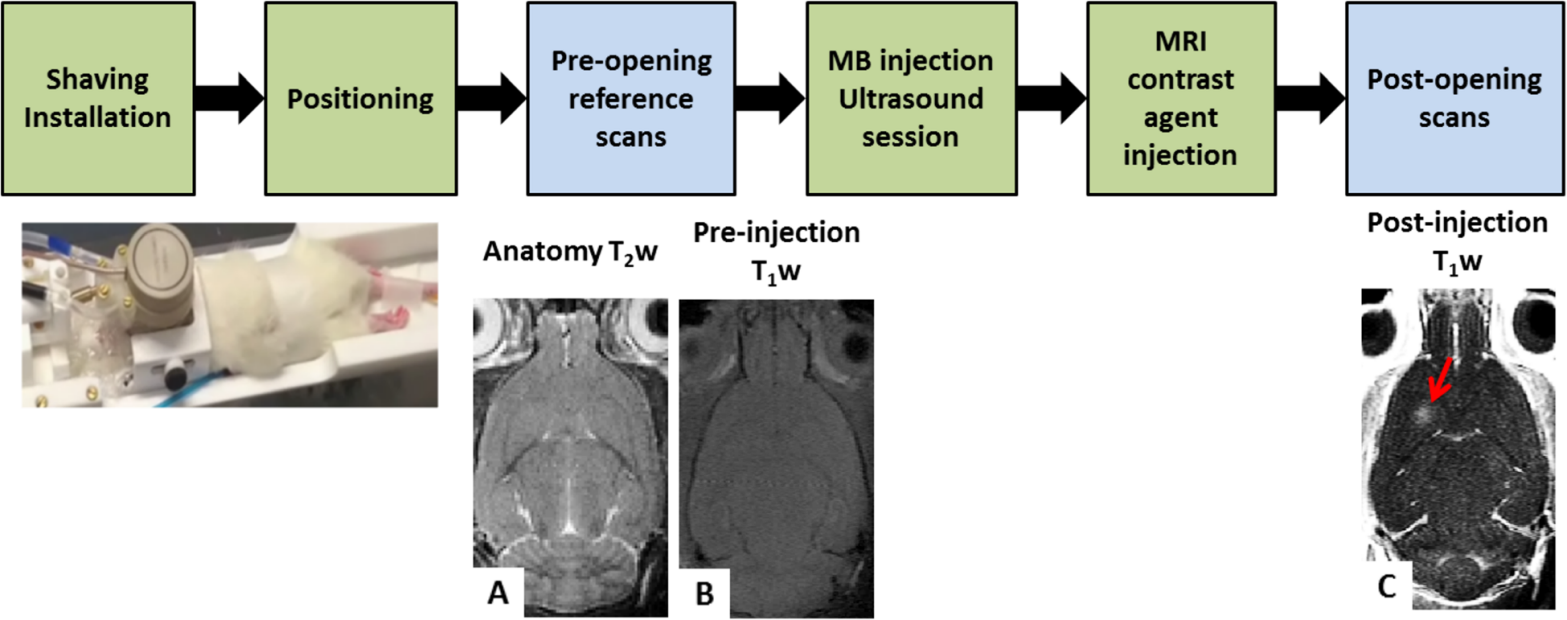
Overview of the BBB permeabilization experiment. The different steps are summed up in the *first row*. The rodents are shaved and installed in the bed under anesthesia, and a catheter is set in the caudal vein. It is then inserted in the magnet where reference scans (*second row*, **a** and **b**) and positioning procedure are performed using ARFI images and the motors. The BBB permeabilization procedure is performed followed by MR contrast agent injection and post-permeabilization MR image acquisition (**c**). MB = microbubbles. The *red arrow* indicates the BBB permeabilization visible by a contrast enhancement on the T_1_-weighted image (**c**)

All animal experiments were performed in accordance with national ethic laws (project authorization number: 12-058, site authorization number: B-91-272-01). Sprague Dawley rats of 120 g (*n* = 10, Janvier, France) and C57Bl/6 (*n* = 1, Janvier, France) mice were used. Their heads were chemically shaved to ensure proper coupling with the transducer. They were anesthetized with isoflurane (1.5–2 %) in a mixture of air and oxygen and then positioned into the bed in prone position (Fig. 3). The ultrasound transducer was coupled to the head via the water balloon and echographic gel. A custom-made catheter (25 G), filled with saline and 10 % heparine to avoid blood clot formation, was inserted in the caudal vein, to inject microbubbles and MRI contrast agent from outside the scanner with minimal dead volume (tubing from Fisher Scientific with an inner diameter of 0.5 mm). The motorized setup was placed in the magnet bore hole with the animal head at the isocenter.

An ARFI image was acquired to localize the ultrasound focal spot (Fig. 2) and could be used to reposition the ultrasound beam in the targeted region of interest. From this starting point, the BBB permeabilization trajectory was defined, including an ultrasound sequence and a mechanical displacement of the transducer if needed. Sonovue® (Bracco, Italy) was administrated via a bolus injection (1.5 × 10^8^ bubbles/mL, 1.6 mL/kg, 2 s) in the catheter and flushed with 100 μL of saline, immediately followed by the sonication along the defined trajectory. Dotarem® (Guerbet, France), a Gd chelate, was then injected (1.6 mL/kg, 2 s) right after the sonications. T_1_w images were acquired to visualize the contrast agent penetration at the expected BBB permeabilization location. Different ultrasound protocols were tested, using the 1.5-MHz single channel transducer. The whole protocol is summarized in Fig. 3.

The in situ acoustic pressure was estimated given the calibration of the transducer in free water and a transmission factor of 0.7 for the skull of 120-g rats (previously measured in a water tank).

### Protocol #1: targeting accuracy in vivo

**Fig. 4:**
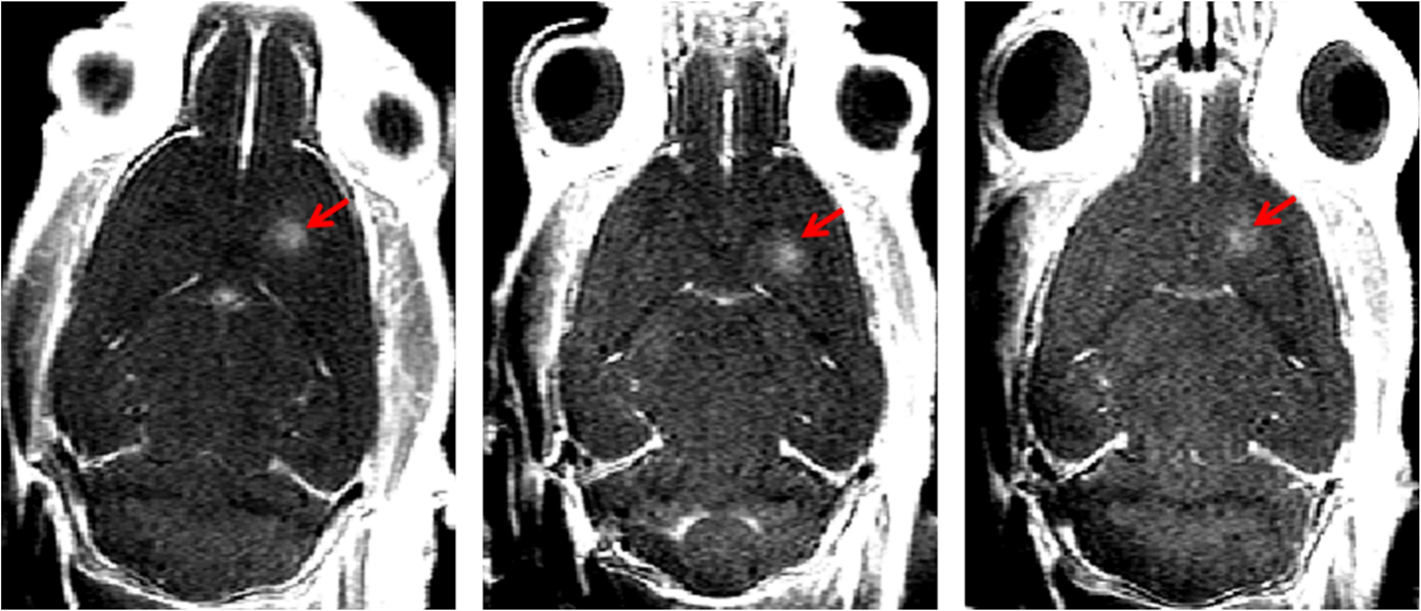
Targeting accuracy in vivo*.* T_1_-weighted images obtained after Gd injection on three different rats on which the same target in the right striatum was chosen. The contrast enhancement is visible only where the BBB has been disrupted (*red arrows*). It appears that the position is reproducible in these different rats

Protocol #1 was aimed at addressing the targeting accuracy in vivo, by targeting the same location in the striatum on 3 different rats (Fig. 4). A focused BBB permeabilization was then performed without moving the transducer with 3-ms ultrasound shots followed by 97-ms pause, repeated during 1 min with a focal acoustic pressure of 0.6 MPa (transducer A). The central position of the opening was then estimated on T_1_w images acquired after the BBB permeabilization for the three rats.

### Protocol #2: BBB disruption along arbitrary trajectories

**Fig. 5:**
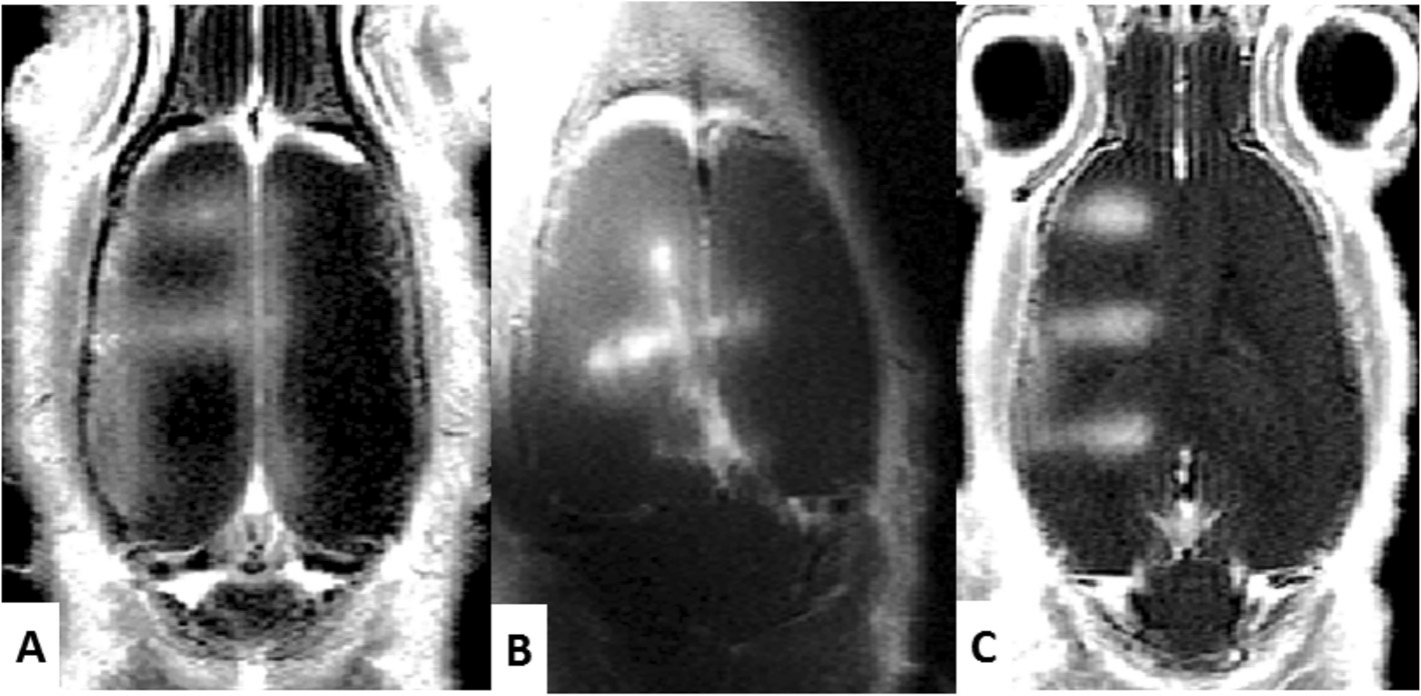
BBB permeabilization along arbitrary trajectories. **a**, **b**, and **c** T_1_-weighted images obtained after Gd injection, showing contrast enhancement at the permeabilization location. They were disrupted along trajectories representing, respectively, the letters “A,” “X,” and “E”

**Fig. 6:**
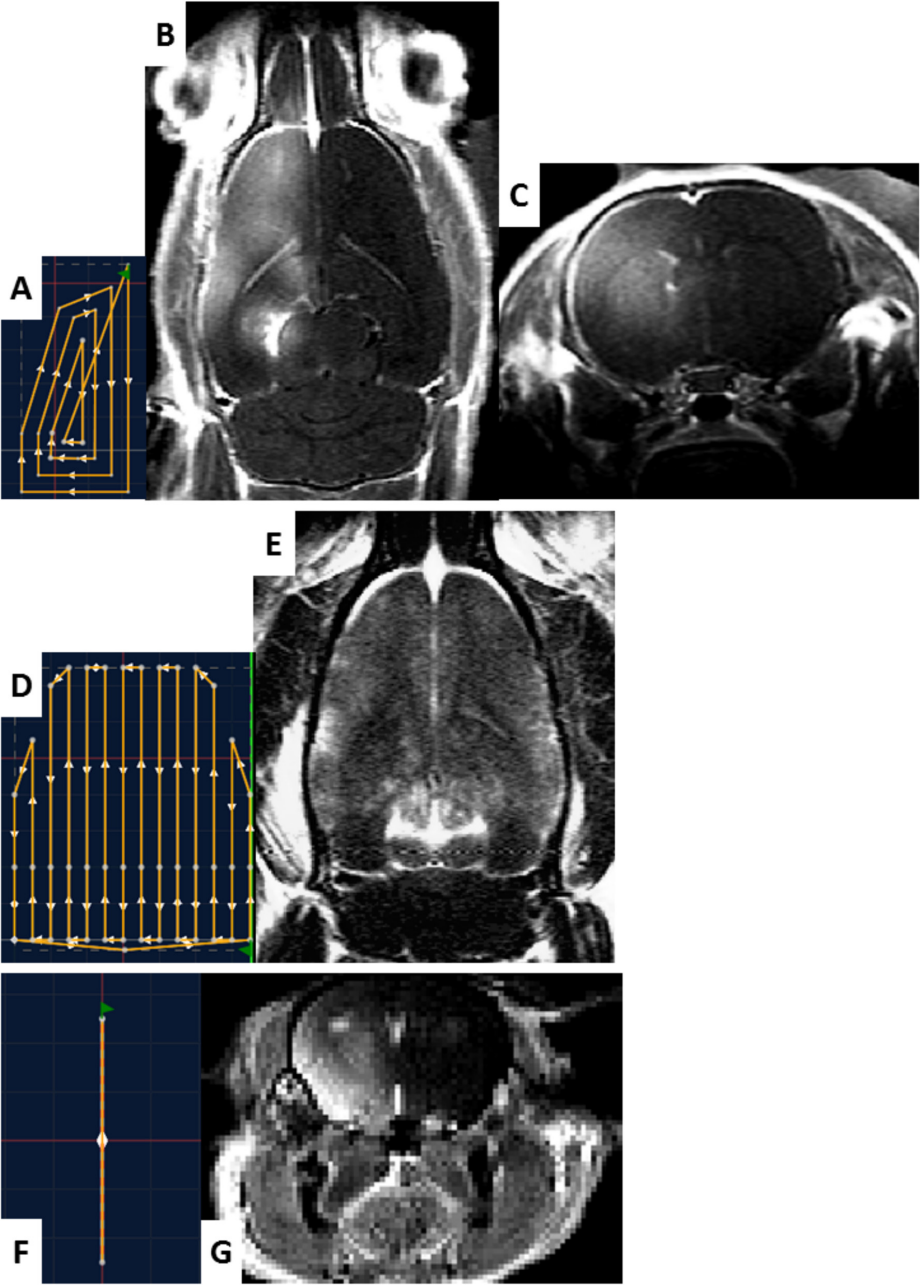
Trajectory drawing and application to hemispheric and global permeabilization. **a** (resp. **d** and **f**) Trajectories which were designed in the control software for hemispheric BBB disruption in rats (resp. global BBB permeabilization in rats and hemispheric BBB disruption in mice). **c** (resp. **g**) Axial T_1_-weighted image obtained after Gd chelate injection, showing contrast enhancement only in the hemisphere which has been disrupted in rat (resp. mice). **b** The corresponding T_1_-weighted image obtained in the same rat in horizontal orientation. **e** T_1_-weighted image obtained in horizontal orientation for the rat with global BBB permeabilization

In protocol #2, several mechanical trajectories were designed to demonstrate the feasibility of BBB permeabilization along arbitrary trajectories. On rats #2.1, #2.2, and #2.3, trajectories with “A,” “X,” and “E” shapes were defined (Fig. 5). On rat #2.4, a trajectory covering the whole left hemisphere was defined (Fig. 6a). For all tested trajectories, continuous sonications were performed. They were repeated 60 times with a 100-ms pause between each execution and a moving speed of 10 mm/s. Experiments were conducted with transducer A. Finally, a trajectory covering the whole brain was designed on rat #2.5 (Fig. 6d). The acoustic pressure was kept constant to 0.6 MPa in situ along the trajectory by adapting the transmitted power thanks to a map of the rat skull transmission factor (data not shown). Transducer B was used for this protocol.

The feasibility of hemispheric BBB permeabilization was also investigated on a C57Bl6 mouse, using the same preparation process and MRI sequences for rats. The sonication pattern was 3-ms sonications every 100 ms, and the transducer was continuously moved along a line over a whole hemisphere during 10 min (Fig. 6f). Dotarem® was then injected to visualize the disruption.

### Protocol #3: influence of acoustic pressure on BBB permeabilization

**Fig. 7:**
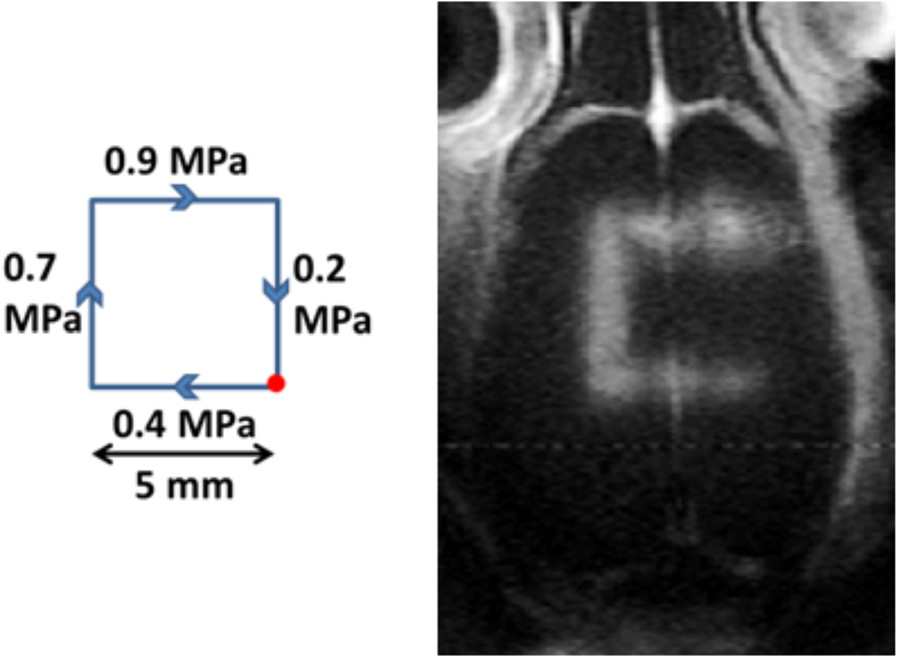
Influence of acoustic pressure on BBB permeabilization. T_1_-weighted image obtained after BBB permeabilization along a square trajectory with different acoustic pressures on each side for rat #3.1 and a scheme of the trajectory used

Protocol #3 aimed at showing the possibility to test different acoustic conditions on the animal during the same session. A square trajectory with 5-mm length was defined on one rat (Fig. 7). Focal acoustic pressure varied on each side of the square: 0.2, 0.4, 0.7, and 0.9 MPa for rat #3.1. The sonications were continuous, repeated 60 times with a 100-ms pause between each execution, and a moving speed of 7 mm/s. The total sonication time was 3 min.

## Results

### Protocol #1: real-time monitoring and targeting accuracy in vivo

Figure 2 shows a typical positioning procedure combining our real-time image processing pipeline and our feedback procedure applied to transducer position. During the first step, an anatomical image (Fig. 2a) was sent to the US console to precisely choose the desired target location of BBB disruption. A first ARFI image was acquired, sent to the console, and processed by the control software to localize the current focal spot (Fig. 2b), which was either automatically detected or could be manually pointed. Once its coordinates have been determined, the motors automatically moved the transducer to the previously defined target position (Fig. 2c). Contrast enhancement on T_1_w images acquired after Dotarem® injection (Fig. 4) confirm BBB permeabilization on three different rats where the same target location in the striatum was defined. The distance between the center of the focal spot and the end of the olfactory bulb was measured in each image. The maximum shift on BBB permeabilization location between the three different rats was 0.5 mm. It has to be compared to the resolution of the ARFI sequence used for positioning (0.5 mm) and the size of the focal spot measured in water tank (FWHM of 1.2 mm).

### Protocol #2: BBB permeabilization along arbitrary trajectories

The images obtained after BBB permeabilization and Dotarem® injection are shown in Figs. 5 and 6. On T_1_w images, Dotarem® reaching brain tissues where the BBB has been disrupted are revealed by the contrast enhancement due to T_1_ shortening of surrounded water molecules. In Fig. 6, a contrast enhancement can be observed 30 min after Dotarem® injection in the whole targeted hemisphere of both rats and mice, while no specific contrast variation is detected in the non-sonicated hemisphere. One can notice that the contrast enhancement in sonicated hemisphere is not perfectly homogenous (Fig. 6b), in particular close to the left ventricle. The designed trajectory (Fig. 6a) could explain this difference, as the segments near the center of the trajectory (corresponding to the position of the ventricle) are more spaced. In the global BBB permeabilization experiment, a homogenous contrast enhancement can be seen on the whole brain, while no specific Gd penetration can be seen in the cerebellum which has not been sonicated (Fig. 6d). Figure 5 also confirms a specific contrast enhancement along the expected BBB permeabilization location only. On post-sonication T_2_w images, no radiological evidence of brain damages was detected.

### Protocol #3: influence of acoustic pressure on BBB permeabilization

A contrast enhancement can be seen along the trajectory, except on the side which experienced the lowest peak negative pressure (0.2 MPa). This confirms a previous result showing a minimum acoustic pressure to be reached for efficient BBB opening [28], estimated to be around 0.4 MPa. We also noted on the T_1_w image that the square exhibits wider permeabilization for increasing acoustic pressure. This result could be explained by assuming that the pressure field has a Gaussian profile, as it is the case in free water. Thus, if the focal pressure is increased, the disruption threshold is reached over a wider portion of the focal spot.

## Discussion

The first results of this work support a successful integration of a head holder consisting of ear bars and a bite bar, a dedicated RF coil, and an 8-channel ultrasound transducer within the 90-mm gradient insert of a 7-T preclinical MRI scanner. The interest of electronic steering in depth is not only to change the depth of focus in the brain but also to adapt the focal length depending on the inflation of the coupling water balloon which can slightly vary from one animal to the other. Furthermore, the use of external motors allows, from outside the magnet, to move the focal point in the brain within a longitudinal (resp. perpendicular) range of 27 mm (resp. 15 mm), with an accuracy estimated in vivo to be better than 0.5 mm. Finally, the design of two different switchable beds offers the possibility to perform BBB permeabilization experiments on either mice or rats with the same setup. It has to be noted that the whole system was designed to be highly modular, both on the hardware side (several compact MR-compatible transducers available, several beds) and on the software side (real-time MR data export and display, dedicated plug-ins for automatic focal spot repositioning).

The system could be further improved by integrating more sensitive and more homogeneous RF coils, by further miniaturizing the transducers or by reducing small mechanical vibrations during scans. It also needs to be noted that the water flow in the balloon was stopped and the transducer/motors were disconnected as often as possible during imaging to avoid artifacts. The coil tuning and matching as well as the shimming in the brain did not change much with the transducer position though.

In the first in vivo experiment, we demonstrate our capability to transiently permeate the BBB at any arbitrary brain location previously chosen on MRI anatomical images, with high reproducibility. As the location of the disruption site can be modified thanks to the motors without taking the animal out of the magnet, the system is time-saving compared to iterative manual positioning.

Moreover, the dedicated Thermoguide® software, which controls the ultrasound shots, allows planning 3D trajectories of BBB disruption with both the mechanical displacement and the electronic beam steering. Such a system should be of great interest to study the dependency of BBB disruption with the targeted brain region, for example, the difference between white and gray matters or the influence of vasculature. Our results demonstrate that this system can achieve delivery of large amounts of MRI contrast agents along virtually any arbitrarily chosen brain areas.

In the second in vivo proof of concept, this system was used for hemispheric BBB disruption, while the other hemisphere was kept as a control, or for BBB disruption over the whole brain. Thus, it offers the unique perspective to deliver therapeutic molecules to large regions of the brain within a preclinical MRI. For example, this could be helpful to perform therapeutic proof of concept studies on pathologies affecting the whole CNS, such as Alzheimer’s disease, for which repeated BBB permeabilization over large regions of the brain have recently shown a great potential [17, 22].

Despite the fact that many features are integrated in the system, the complete BBB opening protocol remains quite complex. First, the intravenous injection of microbubbles in the tail vein still requires human skills and can sometimes be difficult (black mice for instance) or poorly reproducible. The whole process of animal positioning and preparation also remains time-consuming and limits the number of animals that can undergo the protocol. This is the price to pay to ensure the highest level of experimental control. Thus, this system is more suitable for therapeutic proof of concept in rodents and fundamental studies on the BBB opening mechanisms which are still missing before global translation into clinics than for large-group studies.

Our third in vivo application has shown that it is possible to test various acoustic conditions on the same animal during one single session. This ensures a fair comparison of the effect of different ultrasound parameters under the same physiological state. This is very important since studies have shown that some physiological parameters, such as vasoconstriction, play a major role in the efficiency of BBB permeabilization [29]. Our results seem to confirm the existence of a threshold on acoustic pressure below which no penetration of contrast agents is observed, meaning either that the BBB is not disrupted or that the permeability is not enhanced enough to let a significant amount of molecules penetrate into the brain tissues. Although the existence of this threshold was already observed for focal BBB permeabilization [24, 28], this is to our knowledge the first time that it is exhibited for low-pressure continuous sonications together with transducer displacement. The dependence of this pressure threshold with the local acoustic duty cycle during a moving beam should be studied in future studies.

Finally, this system is also suitable for other applications of MR-guided FUS than BBB disruption. Other transducers working at different frequencies can be mounted on the system. For example, low frequencies can be better suited for neurostimulation experiments [37–43], where it will be of great interest to be able to choose a precise stimulation location and to follow in real time the induced neuronal activity with functional MRI [44]. On contrary, high frequencies are more suited for thermal applications (higher thermal deposition) [45], where it is also necessary to precisely target the treatment site and to follow in real time the delivered thermal dose using MR thermometry.

## Conclusions

In this study, we developed a motorized MR-compatible ultrasound system and demonstrated its capabilities to enhance the delivery of large molecules to the CNS either locally or over any large region of the brain. This enables to test several acoustic conditions on one single animal during one BBB disruption session. Reversibly, it can also be used to measure spatial variability of BBB disruption in different brain regions.
